# 

**Published:** 1923-08

**Authors:** 


### PUBLIC HEALTH IN PARLIAMENT.

The following answers have been given for the Minister of Health.

### NATIONAL HEALTH INSURANCE.

Allocation to Voluntary Hospitals.—It is open to any Approved Society having a disposable surplus on valuation to devote the whole or part of such surplus to making payments to hospitals or convalescent homes towards securing the maintenance and treatment therein of members of the Society, and out of the surpluses on the first valuation a sum of £979,000 was set aside for this purpose by certain societies in England in respect of the period from July, 1921, to June, 1926. The selection of the additional benefits to be provided by a society for its members rests with the members themselves, and I do not think it desirable that legislation should be introduced to make it compulsory on every society having a disposable surplus to allocate the whole or part of the surplus to any particular additional benefit.

Panel Doctors.—The Government cannot at this stage undertake that when a settlement has been reached with the doctors an opportunity will be given for the discussion of the new agreement before it comes into operation; but even if no express legislation is necessary, Parliament will have an early opportunity for reviewing the terms of the agreement next Session on the Vote on Account.

Approved Societies (Administration Allowances).—The Minister has received a letter from the Association of Approved Societies to the effect that 4s. 10d. is the minimum sum on which approved societies can effectively carry on their work.

Sickness Benefit (Persons over 70).—The extension of the right to sickness and disablement benefits to persons over seventy would require an increase in the weekly contributions, and would present serious administrative difficulties to Approved Societies in the application of the test of incapacity for work. In view of these considerations, and of the provisions of the Old Age Pensions Acts, legislation is not contemplated.

Benefits (Effect of Unemployment).—The claims for benefits under the National Health Insurance Acts during the year 1922 showed an increase of about 5 per cent. above those for 1921, but this was largely due to an increase in the cost of disablement benefit in accordance with the actuarial expectation. Taking the insured population as a whole there is no evidence that the prevalence of unemployment has adversely affected the claims for benefit under the Acts.

### THE PUBLIC HEALTH.

The Vaccination Acts.—I cannot undertake to introduce legislation for the amendment of the Vaccination Acts during the present Session.

Reheated Milk.—It is expressly provided in the Milk (Special Designations) Order, 1923, that the milk sold as "pasteurised" milk shall not be heated more than once. . . . I am advised that the danger arising to public health from the sale of reheated milk is not such as to justify the issue of special regulations which in present circumstances might result in restricting the supply of milk.

Use of Humane Killer.—I cannot undertake to promote legislation at the present time. It is open to any local authority to adopt the model bye-law providing for the compulsory use of a humane killer, and ninety local authorities have already done so.

### CANCER RESEARCH.

Government Grants.—In addition to the sum of £5,000 expended in grants for work directly upon cancer, radium owned by the Government to the value of over £70,000 is made available by the Council for approved research into the treatment of cancer. The problems of cancer may be solved sooner, and perhaps only through advances in fields of medical science other than the direct study of the disease itself, and advances in the knowledge of cancer are effectively promoted by the grants for research in the more primary parts of the medical sciences.

### LOCAL RATES.

Doubled Since the War.—The amount of local rates paid in England and Wales in 1913-4 was £71,276,000. It is estimated that the corresponding amount for the year 1922-23 was £159,000,000.

### RECENT APPOINTMENTS.

### Medical Officers, Chaplains, etc.

Ash, Walter M., M.B., B.S., D.P.H., Assistant C.M.O.H. and School M.O., Lancashire County Council; Deputy County D.P.H., County Council of Middlesex (London University).

Bland-Sutton, Sir John, F.R.C.S., L.R.C.P., President of the Royal College of Surgeons.

Feldman, W. M., M.R.C.S., L.R.C.P.; Physician to St. Mary's Hospital for Women and Children, Plaistow.

Forrest, A. W., M.D., D.P.H.; M.O.H. Wandsworth, £750 plus £225 bonus.

Hunter, Lieut.-Colonel H., Secretary of Bristol Homœpathic Hospital.

M'Dowall, R. J. Stewart, D.Sc., M.B., Ch.B.; Professor of Physiology, King's College (Edinburgh University).

M'Kendrick, William, M.D., D.P.H., M.O. West Renfrewshire; M.O. Hartlepool (Glasgow University).

Rutherford, Andrew, M.B., Ch.B.; M.O.H. for the Borough of Hawick.

### Public Health and Hospital Appointments.

Bath, E. M., Assistant County Superintendent (Queen Victoria's Jubilee Institute), Surrey.

Bishop, A. M. (L.L.A., St. Andrews); Matron, Jessop Hospital, Sheffield (King's College Hospital).

Cording, E. M., School Nurse, Bristol (Gloucester Infirmary).

Crocker, H. L., School Nurse, Bristol (Bristol Royal Infirmary).

Evans, S. M., Health Visitor, Monmouthshire County Council (Llanelly General Hospital).

Hughes, A., Health Visitor, St. Helen's; Health Visitor Eccles Borough (West Derby Union Infirmary, Walton).

Law. Jessie Davidson; Health Visitor and Clinic Nurse, Heston and Isleworth District Council (Western Infirmary, Glasgow.)

MacInnes, Mary, Matron Gogarburn Children's Institution, Matron Glasgow Eye Infirmary. (Western Infirmary; Glasgow.)

Melliar, E. C., School Nurse, Bristol (Bristol Royal Infirmary).

Ritson, May, Health Visitor and School Nurse, St. Helens; Assistant Health Visitor, Southport County Borough (Alderley Edge Hospital).

Scourfield, M. M., Health Visitor and School Nurse, Hereford City (St. John's Hospital, Wandsworth).

Smith, G. H., Sanitary Inspector, Norwich; Sanitary Inspector Cannock Urban District Council.

Thomas Nellie, Health Visitor, Bangor City (Brownlow Hill Hospital, Liverpool).

Weir, Alexander B.; City Analyst, Aberdeen, £400.

Williams, Mary, Health Visitor and School Nurse, Denbigh County Council (Liverpool Royal Infirmary).

### MICROCOCCI IN CONDENSED MILK.

A special report has been issued by the Food Investigation Board of the Department of Scientific and Industrial Research dealing with studies in sweetened and unsweetened (evaporated) condensed milk made by Dr. William G. Savage and Dr. R. F. Hunwicke. The need for the invariable use of air-tight containers for sweetened condensed milk is emphasised, and has a practical bearing on infant feeding. The tins contain more milk than is consumed by an infant in several days, especially if used, as so often is the case, as a supplementary source of feeding. Although the initial bacterial content may be very low, the mere fact of opening, quite apart from any bacterial additions from outside, induces a vast increase of the bacteria already there. Given the greatest care and success in keeping it free from sources of contamination, this multiplication will still take place. Although micrococci are of little or no significance as a cause of unsoundness in canned fish or marine products, experience has shown that they must be most carefully considered as a possible cause of decomposition in unsweetened condensed milk.

